# Species dynamics in natural bacterial communities over multiple rounds of propagation

**DOI:** 10.1111/eva.13470

**Published:** 2022-10-25

**Authors:** Anneloes E. Groenenboom, Joost van den Heuvel, Bas J. Zwaan, Eddy J. Smid, Sijmen E. Schoustra

**Affiliations:** ^1^ Laboratory of Genetics Wageningen University and Research Wageningen The Netherlands; ^2^ Food Microbiology Wageningen University and Research Wageningen The Netherlands; ^3^ Department of Food Science and Nutrition University of Zambia Lusaka Zambia

**Keywords:** community composition, ecosystem functionality, experimental evolution, long‐term ecology, mabisi, species sorting, traditional fermentation

## Abstract

Our experimental work illustrates how microbial ecosystems can be shaped by selective pressures over long‐term ecological time scales. Natural microbial ecosystems generally consist of various co‐existing species, where community composition describes the frequency at which species or types are present. Overall functionality of the system is achieved by interacting species. Upon short‐term selection, for instance by transfer to a novel environment, community composition and functionality may change in a process referred to as species sorting. Various factors, such as initial community composition and selective pressures from the environment, may influence this change. Mabisi is a traditional fermented food from Zambia that naturally contains a bacterial community of around twenty unique bacterial types. We used six comparable but different natural bacterial Mabisi communities, each split into five identical replicates, for 16 propagation cycles in a novel, common laboratory environment. Composition of the bacterial communities changed upon propagation. The influence of four main factors on community composition, i.e. initial composition (history), impact of the environment (adaptation), changes due to interaction between species and random processes (chance) in species dynamics, was tested using maximum likelihood ratios. Initial community composition seemed to determine the change in community composition, followed by random processes. Interestingly, we observed convergence at the level of ecosystem functionality, which was measured as profiles of metabolic output. This suggests different combinations of species or types can achieve similar eco‐system functionality.

## 1 INTRODUCTION

In many ecosystems various species co‐exist forming species communities. Community composition is defined as the identity and relative abundances of all taxa in the community (Gill et al., 2020). Similar ecosystems have similar species diversities and community composition, for instance in the communities of Darwin's finches on similar islands and communities of cichlid fish in similar African lakes (Grant et al., 1976; Seehausen & Bouton, 1997), showing that similar eco‐systems have similar community composition with regard to species present. Biotic and abiotic factors are thought to generate ecological niches that support multiple species to co‐exist and stabilise eco‐systems (Hardin, 1960). Community composition can change due to selection pressures (Fiegna et al., 2015; Freilich et al., 2011; Gravel et al., 2010; Zaret & Rand, 1971). Selection pressures may shift the balance among the co‐existing species favouring the species which are best adapted to the selection pressure (Lawrence et al., 2012), leading to a process of species sorting (Langenheder & Székely, 2011; Székely & Langenheder, 2014). Key questions include whether species sorting would lead to parallel or divergent change when species communities encounter the same change in environment, when similar species or types are present in ancestral communities, and to what level species sorting is repeatable. Functionality of the eco‐system, which could be defined as the overall output of the system in terms of metabolites, is linked to community composition and may also change upon selection (Eisenhauer et al., 2019; Waldrop et al., 2000; Wolfe & Dutton, 2015).

Here we study how a change of environment can change the community composition of fermenting bacteria in a natural microbial eco‐system derived from Mabisi. Mabisi is traditionally produced in Zambia through spontaneous fermentation of raw milk, resulting in a sour non‐alcoholic product consumed by all age groups. The bacterial community consists of six to ten main species of lactic acid and acetic acid bacteria (Groenenboom et al., 2020; Moonga et al., 2020; Schoustra et al., 2013). Production methods of Mabisi differ per region (Moonga et al., 2019). In most cases, raw milk is filled in a container, either a calabash, bucket or milk can, and left undisturbed for 24–48 h after which it is stirred and consumed. The resulting community is re‐used for the production of the next Mabisi by addition raw cow milk to the containers in which the community is present. These bacterial communities have been co‐cultured up to tens of years or maybe even more. In a food technology context the serial transfer of material is referred to as backslopping (Nout et al., 2005). Mabisi, like other traditional fermented foods, is a means for many small scale processors and entrepreneurs to promote livelihoods and nutrition within a local context (Materia et al., 2021b).

In this experiment, six different original Mabisi samples were used, each split in five replicates and propagated over 16 serial transfers in a common environment. We characterized the samples in terms of the bacterial community composition, i.e. the identity and relative abundances of all operational taxonomic units (OTUs) or bacterial types and functionality (metabolic output) at the start of the experiment and after the repeated transfers. The central question we address is whether initially similar communities will either become more alike (convergence) or less alike (divergence) with respect to the bacterial community composition. Final community composition could be affected by the initial composition (history—OTUs present and their relative abundance) and the selective pressures during the repeated transfers imposed by the environment (change; Travisano et al., 1995). If a new slightly different from the original environment is the main driver of change in community composition, we expect the communities to become more alike. However, should the slight differences in community composition between the six original Mabisi samples be the main driver, we expect community composition to diverge.

By using five replicates of each of the six original Mabisi samples for the repeated transfers, we will assess how repeatable the changes in bacterial community composition and functionality are when starting with communities with slight initial differences in bacterial community composition. This will show whether there would be an optimum community composition in a given environment. Two traits related to community dynamics, metabolic profile and community composition, are measured at the beginning and end of the experiment. We used a custom statistical model to test whether initial community composition and environment were significantly affecting community dynamics and if so, whether this happened according to an additive or interactive scenario. For this we used a log‐likelihood ratio test with multinomial probabilities distributions.

## 2 MATERIALS AND METHODS

### 2.1 Mabisi samples

For these experiments, fermented milk products from Zambia were collected under a collaborative project. This product, called Mabisi, was purchased on the market of Mumbwa, Kaoma and Nangoma in February 2015. In Mumbwa, Mabisi was bought from three producers, and one producer sold two types of Mabisi. This resulted in six Mabisi product samples: four from Mumbwa, one from Kaoma and one from Nangoma (see Appendix S1). (Moonga et al., 2019). This fermentation method involves placing raw milk in a fermentation vessel and allowing it to spontaneously ferment for 48 hours without shaking. Mabisi is a traditional food, where processors use traditional knowledge to process perishable raw milk into a food with prolonged shelf life and improved microbial safety. It is produced in most rural areas in Zambia. Consumers are mostly found in rural towns, yet also consumers in larger cities have interest in this traditional food if available. In this way, mabisi plays an important role in the food system (Materia et al., 2021a; Moonga et al., 2019).

Another study found that neither sampling location nor processor explained significant parts of variation among the bacterial communities. Processing method did explain variation, yet all samples used in this study were produced using the same method (Tonga‐type; Moonga et al., 2020). We thus decided to treat all our bacterial communities uncorrelated and did not perform any analysis using the sampling location as explanatory variable (see Appendix S1).

### 2.2 Repeated propagation cycles

After arrival in the laboratory the bacterial communities were incubated in 30 ml UHT milk (Milbona, Lidl) with 1 ml of Mabisi. The incubation period was 3.5 days at 27°C. The resulting communities (considered T0) were used to inoculate the experiments. Five lines (replicates) were incubated per original Mabisi sample, 750 μl Mabisi in 75 ml UHT milk, resulting in 30 lines. Also two blank UHT milk lines were transferred, but not initially inoculated. Every 3.5 days, 750 μl of the fermented milk was transferred to 75 ml UHT milk, pH was measured and samples (1 ml) were taken for DNA extraction to allow full community profiling. In total 16 transfers were made, resulting in an average of 106 generations, assuming the bacterial diversity could increase a hundred fold after the dilution step during transfer (log(100)/log(2) = 6.64 generations per propagation cycle, gives 6.64 × 16 = 106 generations in total).

### 2.3 DNA extraction

The DNA extraction method was adapted from Ercolini et al. (2001) and Schoustra et al. (2013). All chemicals were obtained from Sigma, unless stated otherwise. For DNA extraction, 1 ml of fermented milk was spun down (2 min, 12,000 RPM), after which the supernatant was removed. The cells were re‐suspended in a mix of 64 μl EDTA (0.5 M), 160 μl Nucleic Lysis Solution (QIAGEN), 5 μl RNAse, 120 μl lysozyme and 40 μl pronase E. After an incubation time of 60 min at 37°C and agitation of 350 RPM, 400 μl ice‐cold ammonium acetate (5 M) was added and the mixture was cooled on ice for 15 min. The mixture was spun down and 750 μl of supernatant was transferred to a tube containing 750 μl phenol. This tube was vortexed and its content spun down (2 min, 12,000 RPM) and 500 μl of supernatant was transferred to a tube containing 500 μl chloroform. This tube was vortexed and its content spun down (2 min, 12,000 RPM) and 400 μl of supernatant was transferred to a tube containing 1 ml 100% ethanol and 40 μl sodium acetate (3 M). This DNA containing tube was left to precipitate at −20°C overnight. The next day, the tube was spun for 20 min at 12000 RPM at 4°C. The supernatant was carefully aspirated, and the DNA pellet was washed by adding 1 ml 70% ethanol. The tube was spun for 10 min at 12,000 RPM at 4°C, after which the supernatant was aspirated again. The DNA pellet was left to dry at room temperature and dissolved in 20 μl 10 mM Tris pH 7.5.

### 2.4 Bacterial community profiling: Bacterial community composition at the level of OTUs

The 36 extracts (6 original Mabisi and 6 × 5 of samples at time point 16) containing DNA from all organisms in the community were sent for bacterial 16S rRNA gene amplicon paired‐end sequencing of the V4 hypervariable region (341F‐785R) on the MiSeq Illumina platform by LGC genomics. Primer sequences are CCTACGGGNGGCWGCAG and GACTACHVGGGTATCTAAKCC (Schoustra et al., 2013).

For further data processing and statistics, the QIIME pipeline (Caporaso et al., 2010), modified from Bik et al (Bik et al., 2016), was used. Paired‐end reads were joined using join_paired_ends.py (with minimum overlap 10 basepairs) after which sequences were trimmed and filtered using cutadapt (v1.11 ‐q 20, ‐m 400) using the known primer sequences. These trimmed sequences were then checked for chimera's, using uchime (v4.2.20, gold database; Edgar et al., 2011), with sequences with a lower chimera score than 0.28 were retained. After these trimming and filtering steps sequences were clustered into operational taxonomic units (OTUs) at 95% similarity threshold after quality check using pick_open_reference_otus.py (‐s 0.1, ‐enable_rev_strand_match TRUE, ‐align_seqs_min_length 75, ‐pick_OTU_similarity 0.95). Taxonomy of the resulting OTUs was assigned to representative sequences using the Greengenes (v13.5) rRNA database. This algorithm gives a representative sequence for an OTU, which were used to perform a local blast using the gold database from uchime. The taxonomy from the top BLAST hit was used for further data processing. Shannon index (H) accounts for both number and evenness of OTUs present and is calculated using:

(1) $$H=-\sum_{i=1}^{s}\left(p_{i}\;\ln\;p_{i}\right)$$

in which *p*_*i*_ is the proportion of reads belonging to category *i*, and *s* is the total number of categories which can be OTUs, species, or genera depending on the level of clustering of the reads. The Shannon index can lie between 0 and ln s (Hutcheson, 1970), depending on the distribution of the categories (evenness).

### 2.5 Bacterial community profiling: metabolic profile

Frozen samples from time points 0 and 16 were defrosted for volatile metabolites profile analyses using GC‐MS using a Trace 1300 Gas Chromatograph with a TriPlus RSH autosampler and an ISQ QD mass spectrometer (all Thermo Fisher). After an incubation of 20 min at 60°C, volatiles were extracted using a SPME fibre (Car/DVB/PDMS, Suppelco) for 20 min at 60°C. Volatiles were desorbed from the fibre for 2 min on a Stabilwax‐ DA‐Crossbond‐Carbowax‐polyethylene‐glycol column (30 m length, 0.25 mmID, 0.5 μm *df*), PTV Split‐less mode (5 min) at 250°C, helium as carrier gas at 1.5 ml/min, GC over temperature at 40°C, 2 min, raised to 240°C (10°C/min) and kept at 240°C for 5 min. Mass spectral data was collected over a range of *m/z* 33–250 in full scan mode with 3.0030 scans/s. Results were analysed with Chromeleon 7.2 CDS Software (ThermoFisher) where 32 signal peaks were identified as volatile metabolites according to their elution time and mass spectral data.

### 2.6 Statistical modelling

To test whether and how the initial community composition impacts the change in community composition upon propagation, we employed a statistical modelling approach using R (R Core Team, 2022). In this model, we related the initial (T0) and end diversity (T16) by a vector of transformation values that link these two communities. The transformation values link T0 and T16 diversity as follows,

(2) $${\mathrm{OTU}}_{i,j,k,T16}={\mathrm{OTU}}_{i,j,T0}w_{i,j,k}/W$$

where OTU_*i*,*j*,*k*,T16_ is the frequency of *i*th OTU from original Mabisi sample *j* at timepoint T16, measured for the *k*th replicate. The transformation values *w*_*i*,*j*,*k*_ determine whether an OTU will increase or decrease. Note that the total sum of the frequencies was scaled by factor W such that the total frequency of OTUs becomes 1. We modeled five scenarios (S1–5); the transformation values were dependent on initial community composition (S1), time (S2), initial community composition + time (S3), the interaction between initial community composition and time (S4), and the effect of the interaction of initial community composition and time + stochasticity (S5). We compare these scenarios to a null hypothesis, where there are no differences between any samples (S0).

We determined the probability of each scenario by calculating the likelihood of sampling an OTU table (*S*_*i*,*j*,*k*,T16_ and *S*_*i*,*j*,T0_) from the OTU table frequencies (OTU_*i*,*j*,*k*,T16_ and OTU_*i*,*j*,T0_). To render it possible to calculate the likelihoods of the distributions, we used a rarefaction in qiime to obtain S (rarefaction.py, ‐m 100). The probability of finding S from a distribution of OTU frequencies is determined using the multinomial distribution function dmultinom() in R (R base) for each of the 36 samples. From all possible OTU models we have taken the one with the highest likelihood, given the modeled scenario. The likelihood would be highly dependent on T16 samples as for each original Mabisi sample there are five times more propagated samples compared to non‐propagated (T0) samples. Therefore, we weighted the log‐likelihood of each of these samples by dividing it by 5. We let *P*(*S*;OTU) be the probability that we sample S out of frequency distribution OTU. Then the log‐likelihood summed over all samples of a scenario is given by

(3) $$L=\sum_{j=1}^{6}\ln\left[P\left(S_{j,T0};\mathrm{OT}U_{j,T0}\right)\right]+\frac{1}{5}\sum_{k=1}^{5}\sum_{j=1}^{6}\ln\left[P\left(S_{j,k,T16};\mathrm{OT}U_{j,k,T16}\right)\right]$$

The frequency distribution with highest likelihood could be determined in all but one scenario, by averaging the frequencies found; i.e. the most likely scenario for frequency is the average frequency. For instance, for S0, where the scenario is that of no difference in distribution between any samples, the most likely OTU distribution is the average over all the OTU frequencies, i.e.,

(4) $${\mathrm{OTU}}_{\mathrm{ML},S0}=\frac{1}{12}\left[\sum_{j=1}^{6}{\mathrm{OTU}}_{j,T0}+\frac{1}{5}\sum_{k=1}^{5}\sum_{j=1}^{6}\mathrm{OT}U_{j,T16}\right]$$

In the second scenario we model the potential effect of initial community composition, without an effect of propagation (S1). The most likely OTU frequency distribution is the average over the OTU tables within a sample coming from one original Mabisi sample.

(5) $${\mathrm{OTU}}_{\mathrm{ML},S1,j}=\frac{1}{2}\left[\mathrm{OT}U_{j,T0}+\frac{1}{5}\sum_{k=1}^{5}\mathrm{OT}U_{j,k,T16}\right]$$

Third, we model only the effect of time point (S2), where the most likely distribution is that of the mean over all OTU frequencies within a timepoint. Therefore, this is

(6) $$\mathrm{OT}U_{\mathrm{ML},S2,t}=\bar{\mathrm{OT}U_{t}}$$

where *t* stands for different timepoints, which can be T0 and T16. Then we modelled the effect of both initial community composition and time, but with similar changes in time for each original Mabisi sample (S3), i.e, *w*_*i*,*1*,*k*_ *= w*_*i*,*2*,*k*_ = …. *w*_*i*,*6*,*k*_.

In this scenario, an evolutionary algorithm was used to find the most likely frequency distribution, as a similar analysis of averaging (Equations (3), (4), (5), (6)) could not lead to the most likely distribution. The aim of this algorithm is to find the most likely values for *w* allowing for interactive effects of time and mabisi samples. For the initial state of *w*, we used the mean values of OTU at T16 divided by T0, scaled in a similar way as in Equation [1]. Subsequently, all the values for OTU at T0 and *w* were mutated by multiplying these by a random number taken from a normal distribution with mean 1 and standard deviation of a uniform distribution that varied between 0 and 0.05. Therefore, neither the steps, nor the step size was equal in every generation. For the obtained values of most likely OTU distribution Equation 2 was calculated and when this likelihood was higher than before, the values obtained in the current run became the newly inherited parameter values for OTU T0, OTU T16 and all values for *w*. This simulation was run for 10,000,000 generations, within which the most likely OTU values showed asymptotic behavior.

Then we modelled the scenario in which initial community composition affected how OTU frequencies changed during propagation (i.e., interaction, S4), which was again estimated by taking the mean of the OTU tables, but now per time point per original Mabisi sample. This was calculated using

(7) $$\mathrm{OT}U_{\mathrm{ML},S4,j,t}=\bar{\mathrm{OT}U_{j,t}}$$

Lastly, when we also allowed for stochastic variation (S5), each most likely OTU table is the sampled table.

Once we had obtained the likelihoods of the most likely distributions, we tested whether initial community composition significantly affected OTU tables by performing a log likely ratio test between S1 and S0. Similarly, we performed such tests between the likelihoods for S0 and S2 (for propagation effect), the interaction between initial community composition and propagation (S3 vs. S4) and lastly whether stochasticity significantly affected the OTU tables by comparing S4 and S5. The value obtained was tested against χ^2^ distribution with difference in parameters as degrees of freedom.

Then we also quantified the relative explaining power for the different factors by calculating McFaddens Pseudo *R* ^2^ (McFadden, 1973). We first calculated these values by

(8) $$R_{\mathrm{Si}}^{2}=1-\log\left(L_{\mathrm{Si}}\right)/\log\left(L_{S0}\right)$$

The maximum *R* ^2^ is obtained from $R_{S5}^{2}$. However, this value is still below 1. To calculate the relative contribution of explanatory power we divided all values of *R* ^2^ by the highest *R* ^2^, $R_{S5}^{2}$, thereby getting *rR* ^2^, the relative McFaddens pseudo *R* ^2^. Lastly, to obtain the added *rR* ^2^ of single factors, such as stochasticity, we had to subtract the value of ${\mathrm{rR}}_{S4}^{2}$. We thereby got the relative contribution for each factor.

### 2.7 PERMANOVA

To verify of our main results are consistent when including multiple time‐points from the series, a second sequencing run was performed including intermediate time points. However, since only Mabisi 4 and Mabisi 6 were additionally sequenced for timepoints 1, 3 and 8 and most but not all mabisi samples at timepoint 8, we performed separate PERMANOVAs on subsets of data, and used a separate likelihood modelling approach only on timepoint 0 and 16 (see below). We tested whether time point and mabisi sample had significant main effects and interactions for all mabisi samples at time point 0 and 16, Mabisi 4 and Mabisi 6 for timepoints 0, 1, 3, 8 and 16 and lastly for all mabisi samples at time points 0, 8 and 16. In all these tests, both main effects and interactions were significant supporting the main finding found in the statistical model. These additional analyses are shown in Appendix S6.

## 3 RESULTS

### 3.1 Bacterial community composition

**FIGURE 1:**
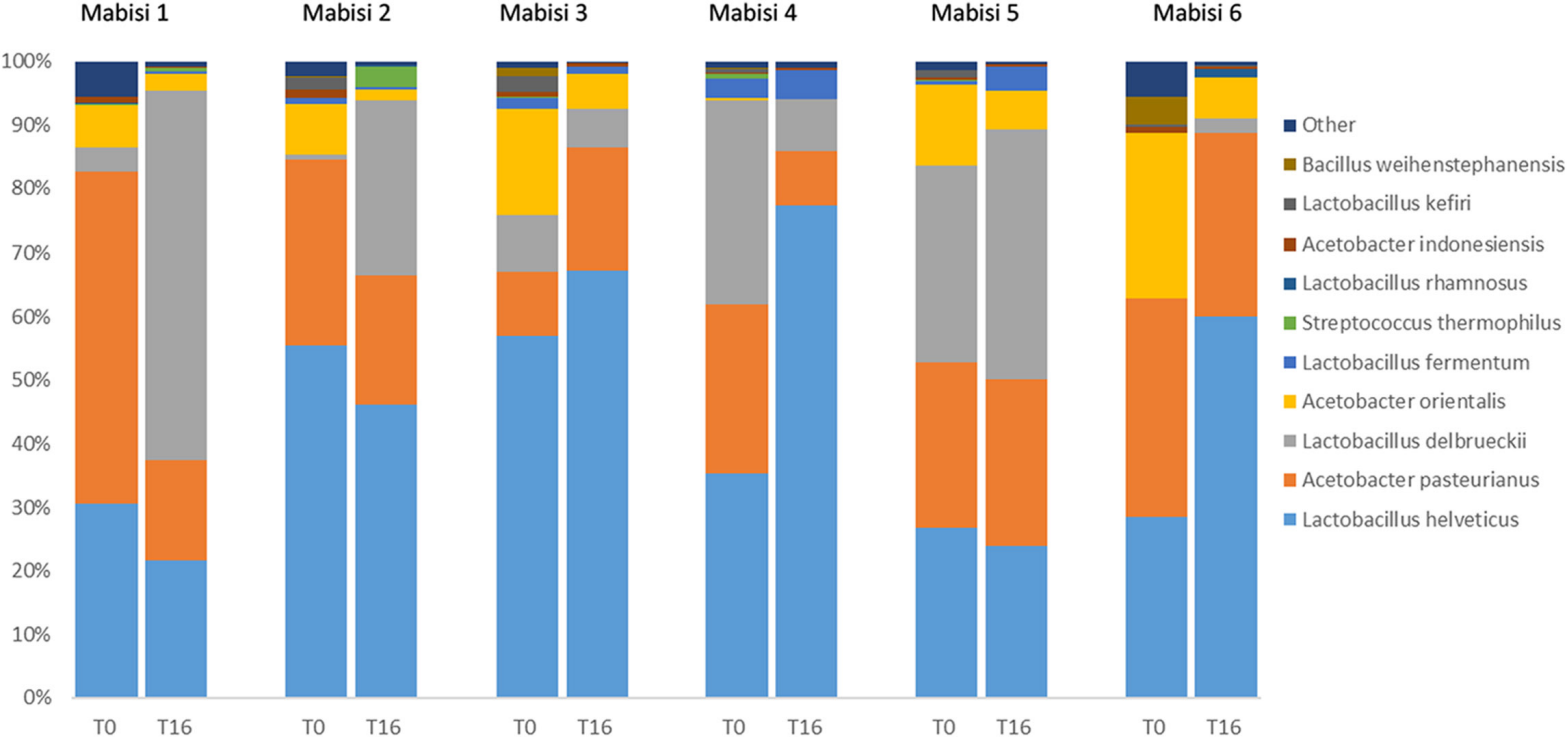
Bacterial community composition of Mabisi samples at time point 0 and the average community composition of the five samples initiated from each Mabisi sample after 16 repeated propagation cycles (time point 16). Results of DNA sequencing in the 16S region, each colour representing an OTU and a legend showing the best match of each OTU to a species. Results are shown as the average for the samples after 16 repeated propagation cycles over five replicates that were propagated independently. Bacterial community structure of all five replicates can be found in Appendix S2.

The experiment was initiated using six initial Mabisi samples. Upon analyses of the microbial communities of these Mabisi samples, a total of 461 different bacterial OTUs were identified based on 16S amplicon sequencing, which blasted as most similar to 47 different species in two main genera, *Lactobacillus* and *Acetobacter*. In two samples, Mabisi 1 and Mabisi 6, 61%–64% of the reads blast as *Acetobacter* species and 30%–35% as *Lactobacillus* species. In the other four samples, which originate from Mumbwa, the microbial communities consisted of about 60%–70% of *Lactobacillus* and 30%–40% *Acetobacter* species. The main species present were *Lactobacillus helveticus*, *Lactobacillus delbrueckii*, *Acetobacter pasteurianus* and *Acetobacter orientalis*. *Lactobacillus fermentum* and *Lactobacillus kefiri* are present in lower amounts and not in all samples (Figure 1). Shannon's diversity index of the samples ranged from 1.75 (Mabisi 4) to 2.11 (Mabisi 3; Appendix S2).

The six Mabisi samples were split in five replicates and inoculated in a new milk environment. After 3.5 days of growth, we transferred 1% of the culture consisting of the mixed bacterial community to fresh milk and repeated the procedure for 16 repeated transfers. After 16 transfers the bacterial community composition was analysed again. The propagated Mabisi communities show differences from the original propagated samples in their bacterial community composition (Appendix S2). On average the relative abundance of OTUs blasted as *Lactobacillus delbrueckii* and *Lactobacillus helveticus* increased while OTUs blasted as *Acetobacter pasteurianus* and *Acetobacter orientalis* decreased in relative abundance. There is variation between groups of propagated lines. In propagated communities originating from Mabisi 4, for example, the abundance of *Lactobacillus delbrueckii* decreases instead of increases. Across all 5 propagated replicates that originated from this Mabisi sample (Appendix S2: Figure S2a), this is a parallel change. Also, for the other replicates originating from one original community a parallel change can be seen. In most cases, except among propagated communities originating from Mabisi 5, the Shannon index was lower after propagation (T16) than before propagation (T0; Appendix S2: Table S2).

**FIGURE 2:**
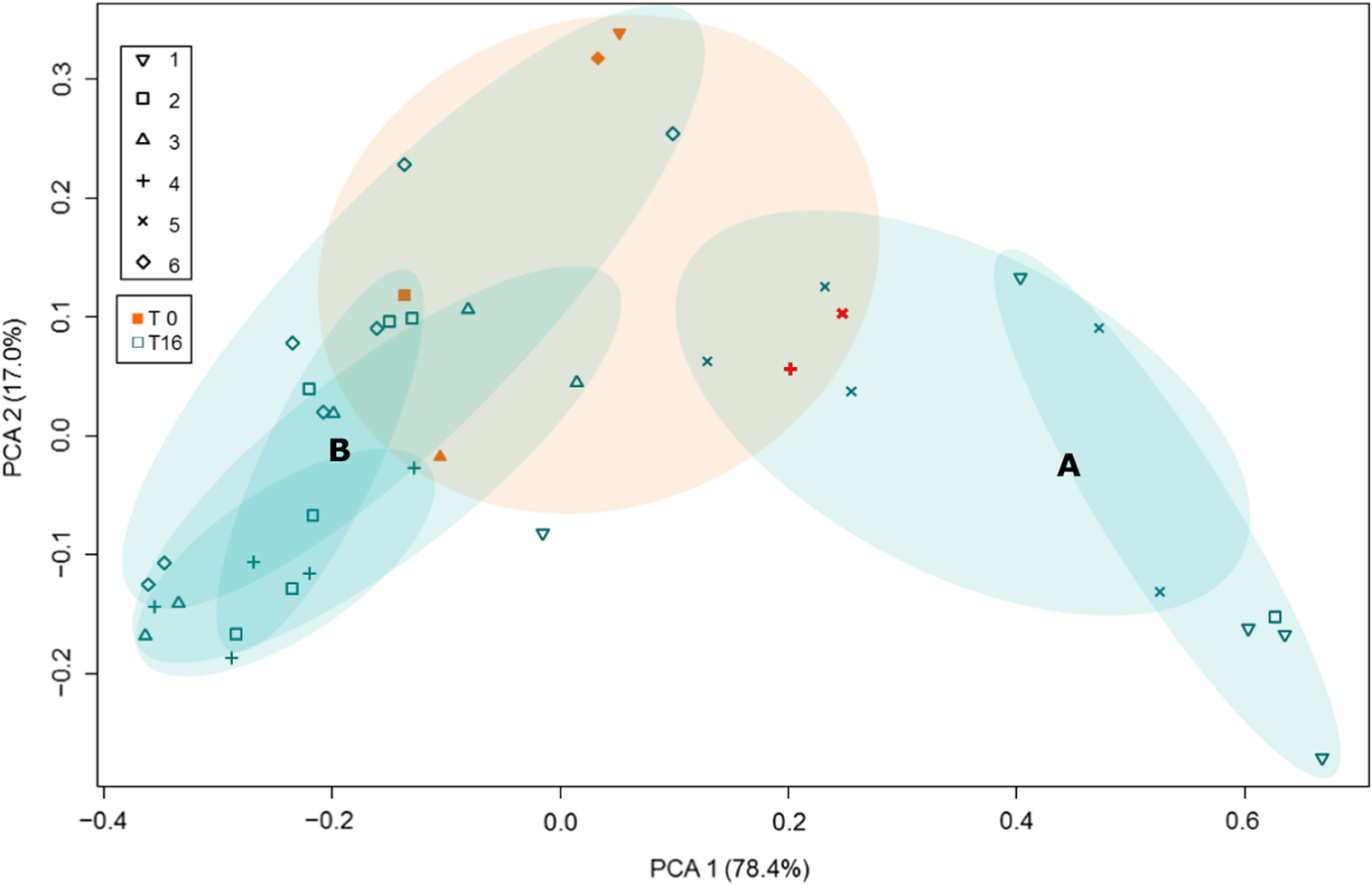
Principle component analyses (PCA) of bacterial community composition of all samples at T0 (orange) and T16 (blue). Shapes represent samples originating from the same original Mabisi sample. Oval orange shades show clustering of T0 samples, blue shades show clustering of T16 samples that had originated from the same T0 sample. Oval shades do not represent results of analysis and are for visual interpretation purposes. Full results of community composition on which this PCA plot is based are in Appendix S2. Two ecological clusters were observed among the T16 samples: Cluster a characterized by a higher relative abundance of *Lactobacillus delbrueckii*, and cluster B characterized by a higher relative abundance of *Lactobacillus helveticus*. PCA analyses were based on OTU tables.

We used a principle component analysis (PCA) to visualize shifts in bacterial community composition after repeated propagation cycles (Figure 2). This analysis shows that bacterial community composition in the five replicates originating from the same original Mabisi sample changed in a similar way. While replicates originating from the same Mabisi sample still show many similarities, the bacterial community composition over all the propagated microbial communities became less similar. Overall, community composition among the propagated communities shows a higher variability than overall community composition among the six starting communities (PERMANOVA, See Appendix S3). Two clusters are formed, representing two ecological states. One with communities originating from Mabisi 1 and 5 (cluster A) and one with communities originating from Mabisi 2, 3, 4 and 6 (cluster B). Compared to cluster B, cluster A is characterized by a decrease in relative abundance of OTUs blasted as *Lactobacillus helveticus* and higher relative abundance of *Lactobacillus delbrueckii*.

### 3.2 Metabolic profiles

**FIGURE 3:**
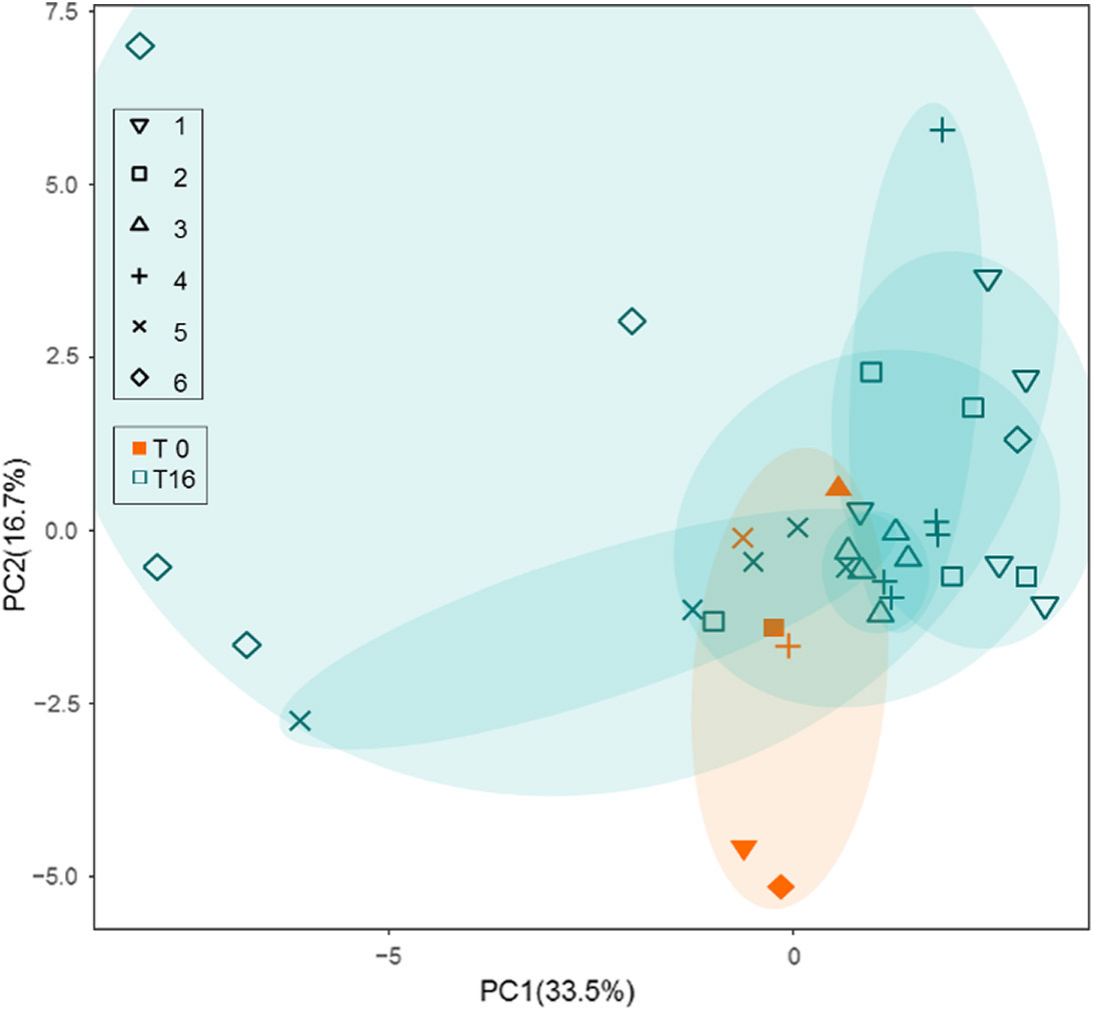
Principle component analyses of volatile compounds of all samples at T0 (orange) and T16 (blue). Similar shapes represent samples originating from the same original Mabisi sample. Oval orange shades show clustering of T0 samples, blue shades show clustering of T16 samples that had originated from the same T0 sample. Oval shades do not represent results of analysis and are for visual interpretation purposes. PCA analyses was based on the 32 volatile compounds detected in the GC‐MS analyses (also see Appendix S4).

For overall metabolic activity, 32 volatiles were analysed as proxy for full metabolic output. Even though non‐volatile metabolites are not analysed, this provides the possibility to compare metabolic activity between the different communities. Most volatiles belonged to the groups of esters, carbolic acids, ketones, and alcohols (Appendix S4). We used a principle component analysis (PCA) to visualize changes in metabolite profiles of communities before and after repeated transfers. In this case, the PCA analysis of the results did not show a clear distinction in clusters (Figure 3). Most samples, except for communities derived from Mabisi 6 show very similar volatile composition which is not, or only slightly, different from the volatile composition at T0. Other community characteristics such as pH, phase separation, and product thickness also did not show a directional change (see Appendix S5 for pH trajectories).

### 3.3 Mechanisms that drive changes in bacterial community composition

Changes in bacterial community composition after repeated propagation cycles can be due to four main factors: initial community composition, selection imposed by the environment, change caused by interaction between species (OTUs) and random processes in species dynamics. To statistically test which factor has the biggest influence on the community structure, we developed a maximum likelihood approach where we add levels of complexity to a community composition model. Using the community composition of the 36 samples we modelled six different scenarios and calculated the log likelihood (Table 1). Visual representation of this step‐wise model is in Appendix S6.

**TABLE 1:** Log likelihood of scenarios what factors affect bacterial community composition

Scenario	Description	Log likelihood
S0 All equal	No initial differences, No diversity changes over time	−562.18
S1 Initial community composition	Initial differences, No diversity changes over time	−364.16
S2 Time	No initial differences, Equal diversity changes over time	−511.24
S3 Initial community composition + time	Initial differences, Equal diversity changes over time	−310.83
S4 Initial community composition × time	Initial differences, Different diversity changes over time	−231.73
S5 In. comm comp. × time + stochasticity	All samples at T16 a different distribution	−134.35

*Note*: The description of the scenarios and log likelihood are listed. OTU cutoff 0.02, likelihood of time point 16 samples averaged. Input = rarefaction_100_1.Biom. 100,000 iterations for estimating S3.

Every step increases the log likelihood (Table 2). Allowing differences between the six initial samples (S1, Initial community composition) explains most of the variation (46.5%) and significantly affected community composition (χ^2^_*df* = 60_ = 396.03, *p* < 0.001, Table 2). While time also significantly affected community composition (12.0% of the variation explained, χ^2^_*df* = 12_ = 101.87, *p* < 0.001), allowing for interactions between Mabisi sample and time points (Initial community composition × Time) explains 18.6% more of the variation in comparison to without interaction (Initial community composition + Time, χ^2^_*df* = 6_ = 158.20, *p* < 0.001). The addition of stochasticity (Initial community composition × Time + Stochasticity) resulted in explaining 100% of the variation, as all possible sources of variation are incorporated, however due to the many parameters included in this model (*df* = 288, Table 2), the addition of stochasticity did not significantly explain more of the OTU table variation (*p* = 1.0).

**TABLE 2:** Statistics of maximum likelihood approach using likelihood ratio tests

Scenarios tested	LR test statistic	Δ*df*	*p*	Rel. McF pseudo *R* ^2^
S1 vs. S0	396.03	60	0	46.5%
S2 vs. S0	101.87	12	6.7e‐16	12.0%
S4 vs. S3	158.20	6	9.8e‐10	18.6%
S5 vs. S4	194.75	288	1.000	22.9%

*Note*: The log likelihood ratio test statistic (2*ΔLL), number of degrees of freedom and *p* value (from chi square distribution) and relative McFadden pseudo *R* ^2^ are listed. OTU cutoff 0.02, likelihood of time point 16 samples averaged. Input = rarefaction_100_1.Biom. 100,000 iterations for estimating S3.

## 4 DISCUSSION

We used natural microbial communities from six Mabisi samples from Zambia as starting points for 16 propagation cycles into a novel environment, splitting each original sample into five replicates. At the start and end of the propagations, we measured bacterial community composition and metabolic profiles, asking whether the composition of these microbial communities would diverge or converge upon propagation in a common environment.

At the genus level, the six original microbial communities show much similarity, however, when comparing the communities at the level of OTUs and corresponding species as which then are blasted, differences between the communities can be observed (Appendix S2: Figure S2b). The level of similarity at the genus level, in combination with a variation in OTUs, made the different bacterial communities a suitable starting point to study potential change in bacterial community composition upon repeated propagation cycles. The original communities harboured similar species (although each species could be present at a different frequency) and thus contained the potential for community composition to converge. The environment used for the repeated propagation cycles—full fat milk in the laboratory—is simpler than the Mabisi environment the bacterial communities originated from. The new environment might have been favorable for only those bacteria that were most fit for this specific environment. This potentially caused a loss in the number of species/OTUs towards retaining only a few OTUs in the microbial community. This is, however, not what we observed. The laboratory milk environment appeared to provide enough niches to the bacterial species to maintain diversity in OTUs.

Our results show that upon propagation in the new environment, the bacterial community composition changed. After 16 repeated propagation cycles two clusters emerged among our propagated communities (Figure 2). The two ecological clusters were characterized by the fact that OTUs classified as either *Lactobacillus delbrueckii* or *Lactobacillus helveticus* was the most abundant species. This implies these two types share the same ecological niche and seem to be interchanging their functionality. What functional properties underlie this interchanging of functionality is subject of further study, which could be implemented using metagenomic sequencing. The present study used 16S amplicon sequencing of the V3–V4 region to characterize the bacterial communities at the level of differences in OTUs. While we blasted these to a database to show to what species these OTUs could belong, our analysis does not allow to fully nor reliably identify at species level. As subspecies are substantially different in their biology and functionality, grouping bacteria by their species level is not detailed enough to determine their function in a certain environment, let alone to detect variations that exist within the same species with respect to their functional properties (Salvetti et al., 2018; Wittouck et al., 2019). Therefore, based on this study, we are unable to elaborate on what functional differences between *Lactobacillus delbruecki* and *Lactobacillus helveticus* may drive the ecological clustering. Further, the natural community used in this experiment very likely contained taxa from other domains (Moonga et al., 2019). The possible interactions between taxa, such as with yeasts, fungi, or bacteriophages, can also give more insight in the microorganisms present to a subspecies or perhaps even lineage level.

Our results suggest that the change in bacterial community composition towards one of the two ecological states is dependent on the original Mabisi bacterial community. Predicting which cluster the community would belong to after repeated propagation cycles does not seem straightforward. For example, original Mabisi 4 and original Mabisi 5 show clear similarities (Figure 2 and Appendix S2: Figure S2a), while the bacterial communities after 16 propagation cycles belong to different clusters.

This dependence on the composition of the original bacterial community was apparent from our maximum likelihood based tests on what factors shape the community composition of propagated communities. The environment was found to have the least influence on the change in community composition of the four tested mechanisms. It is interesting to see that allowing interactions between initial community composition and time explained an extra 18.6% of the variation, compared to the additive effect of initial community composition and time. This indicates that in one propagated lineage a certain OTU increased, while in another the same OTU decreased. We therefore hypothesise that biotic interactions within the community have a bigger influence on the fitness of a certain OTU than the selection of the abiotic environment (Dunson & Travis, 1991).

We had expected that the increased differences in the bacterial community composition would translate into increased differences in metabolic activity (Lawrence et al., 2012; Waldrop & Firestone, 2006). However, in contrast to community composition, the metabolic profiles of the propagated communities did not show two clusters representing the two ecological states. The metabolites produced in a microbial community might thus be more dependent on the environment than on initial and current community composition. The metabolic pathways resulting in the formation of the volatiles measured might be either present in species/OTUs which are represented in both ecological states or be carried by different species but expressed in a similar way. Also, in pH, phase separation and product thickness no directed change during the repeated propagation cycles were found (Appendix S5). We conclude that despite clear differences between the community composition after repeated propagation cycles, the metabolic functionality, and potentially the transcriptomic profile, of the communities as a whole remained similar. This makes that community metabolism and community composition at the level of species are not directly linked. New environments can cause communities to change in composition and function. These changes are influenced by adaptation, chance, and evolutionary history (Travisano et al., 1995). Trait that are strongly related to fitness (such as bacterial growth rate) are more influenced by history, while traits that are weakly related to fitness (such as cell size) are more influenced by environment and chance (Travisano et al., 1995). Community composition was more influenced by the initial community composition (evolutionary history), while for metabolic profile this was not the case.

This experiment can also be seen as an analogy to an experimental evolution with one species starting with standing variation (Prezeworski et al., 2005; Teotónio et al., 2009). In our case, however, we study the sorting of species rather than the sorting of genotypes. Due to selection pressures, one or a few individuals with the highest fitness can be selected in an experiment with standing genetic variation. In case of communities, we cannot speak about fitness of the individual, as the community does not reproduce as one organism, but rather consists of multiple organisms which all reproduce on their own and therefore have their own fitness (de Vos et al., 2018). However, although we cannot use fitness as defined for individual genotypes within a species, we can study how whole communities may adapt to a new environment. Although species are often studied in isolation, in nature they interact with many other organisms. Therefore, this study is focussed on the dynamics of whole communities to complement findings of studies focussing on individuals.

The propagation of bacterial communities into fresh medium was repeated 16 times, amounting to around 100 cell divisions or generations. While novel mutations could arise during our experiment, these are not expected to be frequent nor to have a large impact on community composition due to the limited number of generations. A much longer selection experiment may combine factors of species sorting, which occurs at an ecological time‐scale, with effects of novel mutations that may lead to increase in abundance of some species (Kato & Watanabe, 2010).

## 5 CONCLUSION

When placed in a new (laboratory) environment, different natural bacterial communities from Mabisi maintain their diversity and did not show simple convergent change towards an eroded microbial community with only a few species. Even though the bacterial community composition from the original Mabisi communities seem very similar, small differences made that the final community composition differed between samples from different origin. These changes were parallel in all 5 replicates of the same original Mabisi community. Initial diversity and interactions were determining factors in community composition. However, final composition could not be predicted by initial composition. Despite the changes in community composition, a directed change in function was not found. This suggests that different groups of bacteria might have the same function in this system.

We observed that upon repeated cycles of propagation bacterial community composition was highly dependent on initial composition and to a lesser extent depended on changes caused by the new environment—clean bottles in the laboratory rather than a calabash used by traditional processors. The reproducibility in the way the composition of these communities changed in their new environment opens a door for further research towards finding specific causes within the initial community for the specific compositional dynamics we observed. This would be an important step towards predicting community structure and function in novel environments. Finally, our work illustrates that (traditional) fermented foods are very suitable as tractable systems to study general ecological principles (Alekseeva et al., 2021; Wolfe & Dutton, 2015).

## FUNDING INFORMATION

This work was funded through grants to SES from the Netherlands Organisation of Scientific Research, section Research for Development (NWO‐WOTRO), the Integrated Research Fund of Wageningen University (INREF) and a Marie Curie Fellowship.

## CONFLICT OF INTEREST

The authors declare no conflicts exist.

## Supporting information


Appendix S1–S6.

## Data Availability

All data are available through the 4TU depository (https://doi.org/10.4121/20750437).
