# Supplementary material for: Species dynamics in natural bacterial communities over multiple rounds of propagation
Source: Evol Appl. 2022 Oct 25;15(11):1766–75. doi: 10.1111/eva.13470 (PMC9679247; doi:10.1111/eva.13470)
Supplement: Supplementary file 1 — Appendix S1–S6. [file EVA-15-1766-s001.docx]

**Supplementary material**

**Species dynamics in natural bacterial communities over multiple rounds of propagation**

**Appendix S1.** **Description of the Mabisi samples used in this study**

All mabisi samples were obtained at three rural towns in Zambia from processors selling mabisi at a local market (Photo S1, Map S1). In all cases, the processing of Mabisi was done using the Tonga-type method of processing (Moonga et al., 2019). This fermentation method involves placing raw milk in a fermentation vessel and allowing it to spontaneously ferment for 48 hours without shaking. Mabisi is a traditional food, where processors use traditional knowledge to process perishable raw milk into a food with prolonged shelf life and improved microbial safety. It is produced in most rural areas in Zambia. Consumers are mostly found in rural towns, yet also consumers in larger cities have interest in this traditional food if available. In this way, Mabisi plays an important role in the food system (Materia, Linnemann, Smid, & Schoustra, 2021; Moonga et al., 2019).

All samples used in this study were collected in February 2015. In Mumbwa, two samples were collected from the same processor who had prepared two different (independent) processing batches. We had intended to perform analysis on the outcome of the selection experiment based on where the original samples had originated from. However, after we conducted our study, we had performed a large country-wide survey on Mabisi processing during which we collected over 170 Mabisi samples to profile their bacterial community composition. This study is published as Moonga, H. B. et al. (2020). Composition and diversity of natural bacterial communities in Mabisi, a traditionally fermented milk. Frontiers in Microbiology, 11, 1816. We found that sampling location nor processor explained significant parts of variation among the bacterial communities. Processing method did explain variation, yet all samples used in this study were produced using the same method (Tonga-type). We thus decided to treat all our bacterial communities uncorrelated and did not perform any analysis using the sampling location. (Moonga et al., 2020).

We treated all our samples as harboring independent Mabisi microbial communities.

| Sample# | Sampling location | Processor | Mabisi processing method | Sampling date |
| --- | --- | --- | --- | --- |
| 1 | Nangoma | 5 | Tonga-type | 22 Feb 2015 |
| 2 | Mumbwa | 1 | Tonga-type | 20 Feb 2015 |
| 3 | Mumbwa | 1 | Tonga-type | 20 Feb 2015 |
| 4 | Mumbwa | 2 | Tonga-type | 20 Feb 2015 |
| 5 | Mumbwa | 3 | Tonga-type | 20 Feb 2015 |
| 6 | Kaoma | 4 | Tonga-type | 21 Feb 2015 |


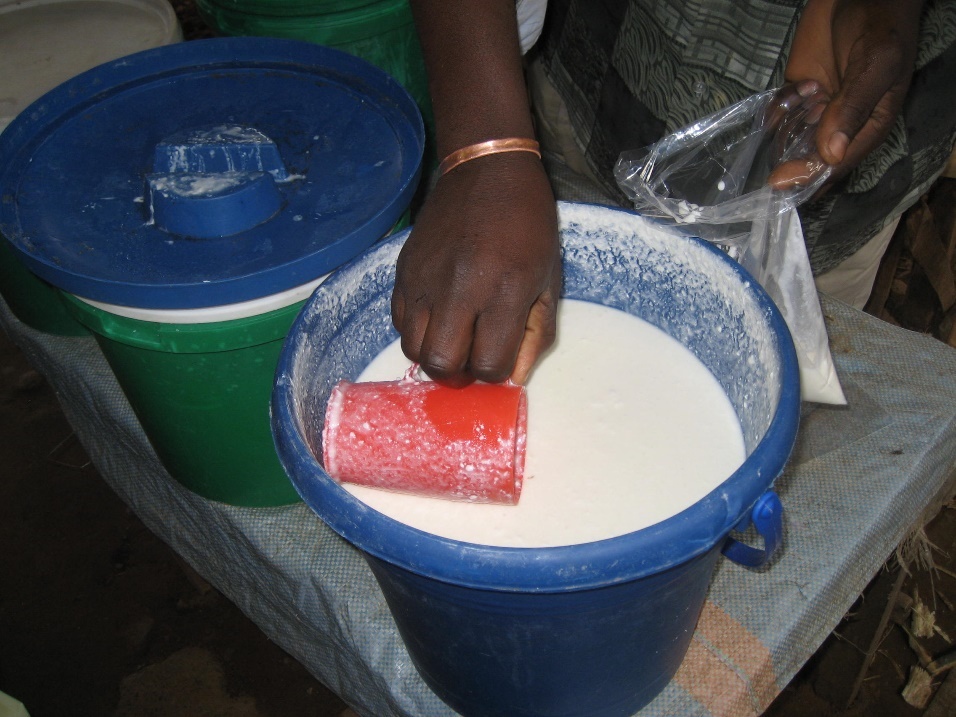


**Photo S1.** Local sales of Mabisi in Mumbwa. Mabisi is prepared at home by a local processor using a plastic bucket. Sales is per cup, which is transferred to a plastic bag. Picture by Sijmen Schoustra.


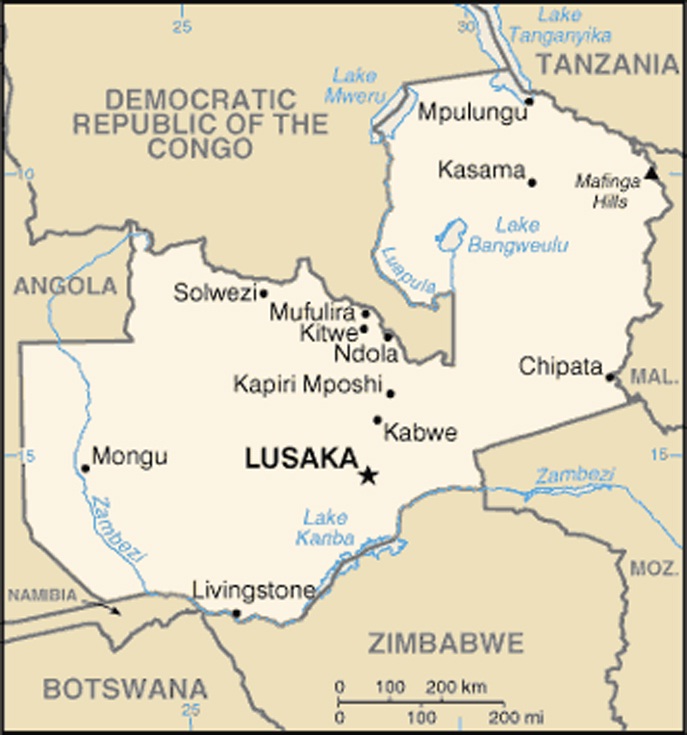


Mumbwa

Nangoma

Kaoma

Map S1. Map showing the sampling locations of for the samples used in this study.

**Appendix S2: Community composition of all initial and propagated communities.**

**Supplemental Figure S2A** Community composition on showing OTUs with corresponding blast identity at species level of all Mabisi samples at time point 0 and propagated samples at time point 16. Different colours indicate different species which could consist of multiple OTUs, see figure S1B below. Vertical axes indicate percentage of total reads within the sample. The nine species with highest abundance are indicated in the legend. The aim of the figure is to show the diversity found in the microbial communities of Mabisi in terms of species variation. For specific abundances of species and OTU, please refer to the sequencing data provided with this publication.

**Supplemental Figure S2B Initial bacterial species diversity of natural communities from mabisi.** The aim of the figure is to show the diversity found in the microbial communities of Mabisi in terms of genus and species variation. Colours differentiate between the four most abundant genera: *Lactobacillus* (green), *Acetobacter* (blue) *Bacillus* (yellow) and *Streptococcus* (red). Shades of the colour indicate different species within that genera, which can consist of different OTUs. Shades of grey represent different species of other genera.

**Supplement Table S2** Shannon index as measure for bacterial diversity on OTU level of all six Mabisi samples, at the beginning of the transfers (T0) and the average after 16 transfers (T16).

|  | T0 | T16 (average) |
| --- | --- | --- |
| 1 | 1.92 | 1.48 |
| 2 | 2.00 | 1.84 |
| 3 | 2.11 | 1.86 |
| 4 | 1.75 | 1.45 |
| 5 | 1.85 | 1.95 |
| 6 | 2.03 | 1.75 |

**Appendix S3: Comparison T0 and T16 and first and second sequencing runs using PERMANOVA and PCA**

Initially a first sequencing run was performed on all samples at timepoints 0 and 16. A likelihood test was used to test whether initial sample composition alters how time affect the change in the communities. The test indicated that there was a significant interaction between sample and time for these two timepoints. A second sequencing run was performed including intermediate time points of two selected series of lineages – those derived from Mabisi 4 and Mabisi 6. However, since only Mabisi 4 and Mabisi 6 were additionally sequenced for timepoints 1,3 and 8 and most but not all mabisi samples at timepoint 8, we performed separate PERMANOVAs on subsets of data, and used a separate likelihood modelling approach only on timepoint 0 and 16 (see below).

First, we tested whether time point and mabisi sample had significant main effects and interactions for all mabisi samples at time point 0 and 16. Indeed both main effects significantly affected the community compositions, as well as the interaction, which was significant (Table S3A, Figure S3A). This indicates that as described above, the community change was different for the different mabisi samples. Secondly, a similar analysis was performed, but now with the addition of the samples at timepoint 8, which were sequenced in the second sequencing run. Again, both main effects and interaction were significant, validating our main conclusion that the direction of change of the community composition was dependent on the initial community (Table S3B, Figure S3). Lastly, for Mabisi 4 and Mabisi 6 we also sequenced timepoint 1 and 3 and therefore performed another separate PERMANOVA and PCA. This again indicated that both main effects and the interaction were significant, further substantiating the main conclusion that the change in community composition in time is dependent on the initial community composition (Table S3C, Figure S3C).

Table S3A. PERMANOVA table for all samples at timepoints 0 and 16. Analysis was performed using the adonis() function with default settings.

| Terms | Df | SS | MS | F | R^2^ | P |
| --- | --- | --- | --- | --- | --- | --- |
| Timepoints | 1 | 0.245 | 0.245 | 6.586 | 0.065 | 0.004 |
| Samples | 5 | 2.217 | 0.443 | 11.945 | 0.593 | 0.001 |
| Interaction | 5 | 0.463 | 0.092 | 2.491 | 0.124 | 0.005 |
| Residuals | 22 | 0.817 | 0.037 |  | 0.218 |  |
| Total | 33 | 3.741 |  |  | 1.000 |  |

Table S3B. PERMANOVA table for all samples at timepoints 0, 8 and 16. Analysis was performed using the adonis() function with default settings.

| Terms | Df | SS | MS | F | R^2^ | P |
| --- | --- | --- | --- | --- | --- | --- |
| Timepoints | 2 | 1.837 | 0.918 | 21.271 | 0.279 | 0.001 |
| Samples | 5 | 1.627 | 0.325 | 7.537 | 0.248 | 0.001 |
| Interaction | 10 | 0.821 | 0.082 | 1.902 | 0.125 | 0.022 |
| Residuals | 53 | 2.289 | 0.043 |  | 0.348 |  |
| Total | 70 | 6.574 |  |  | 1.000 |  |

Table S3C. PERMANOVA table for timepoints 0, 1, 3, 8 and 16 and samples Mabisi 4 and Mabisi 6. Analysis was performed using the adonis() function with default settings.

| Terms | Df | SS | MS | F | R^2^ | P |
| --- | --- | --- | --- | --- | --- | --- |
| Timepoints | 4 | 0.573 | 0.143 | 8.225 | 0.329 | 0.001 |
| Samples | 1 | 0.344 | 0.344 | 19.762 | 0.198 | 0.001 |
| Interaction | 4 | 0.216 | 0.054 | 3.094 | 0.124 | 0.005 |
| Residuals | 35 | 0.610 | 0.017 |  | 0.350 |  |
| Total | 44 | 1.742 |  |  | 1.000 |  |


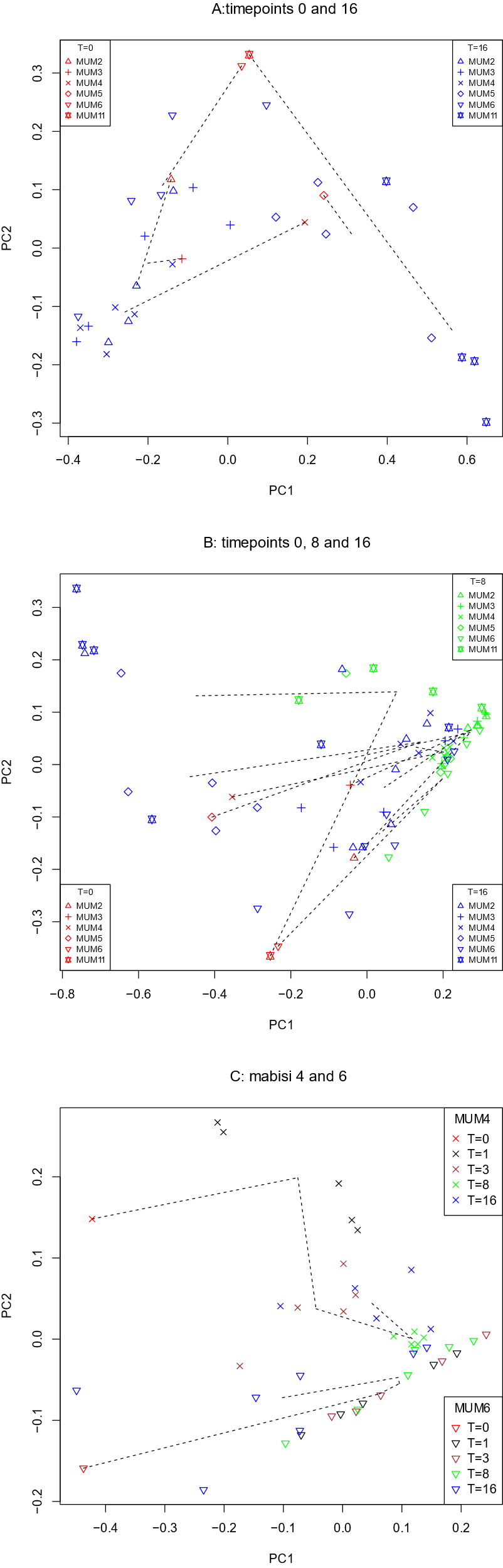


Figure S3. PCA plots of different sets of samples sequenced in the first and second sequencing run. X and Y axis show the values of each sample on the first and second principle component respectively. A) Timepoints 0 and 16 for all samples. Red color indicates timepoint 0, blue 16. Different symbols indicate different mabisi samples. Lines indicate how average of samples change between timepoints. B) Timepoints 0, 8 and 16 for all samples. Red color indicates timepoint 0, green 8, blue 16. Different symbols indicate different mabisi samples. Lines indicate how average of samples change between timepoints. C) Timepoints 0, 1, 3, 8 and 16 for samples Mabisi 4 and Mabisi 6. Different colors indicate the different timepoints, the symbols the different samples.

**Appendix S4. Functional properties of communities as measured by the metabolic profile.**

**Supplemental Table S3 Volatile compounds as proxy for metabolic activity.** Normalised peak area of 32 analysed volatile compounds in 6 initial mabisi samples and 30 (6*5 replicates) samples after repeated propagation cycles.

**Appendix S5. pH trajectories**

| pH |  |  |
| --- | --- | --- |
|  |  |  |
|  |  |  |
|  | Time (transfer) | |

**Supplemental Figure S5**. The graphs show the final pH at the end of each growth cycle over the 16 transfers of the selection experiment. Graphs each have five (mostly overlapping) lines showing all 5 replicates. All pH measures were single measurements for each lineage, hence no error bars are shown.

**Appendix S6. Maximum likelihood analysis on mechanisms that drive changes in community composition**


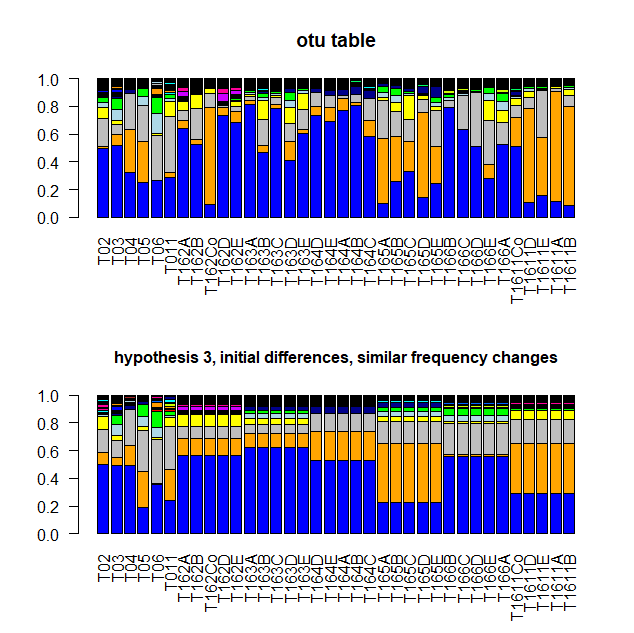

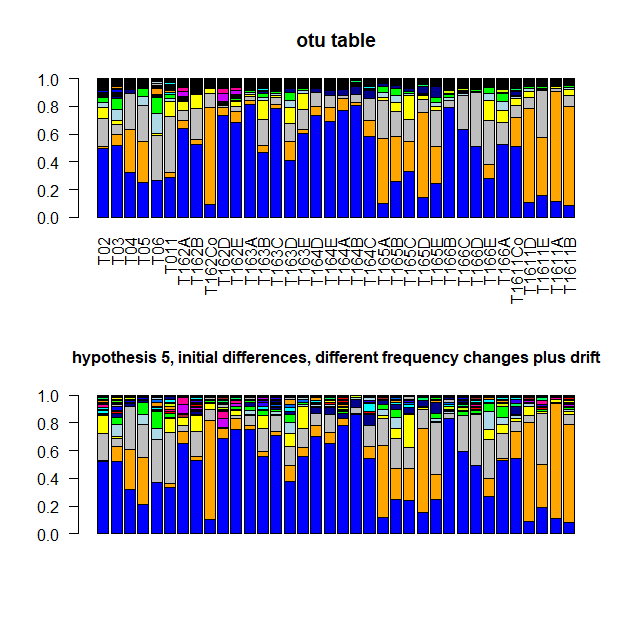

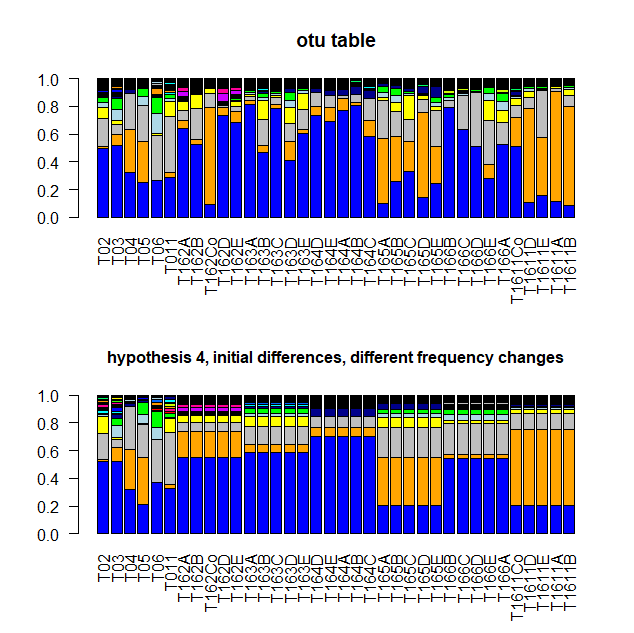

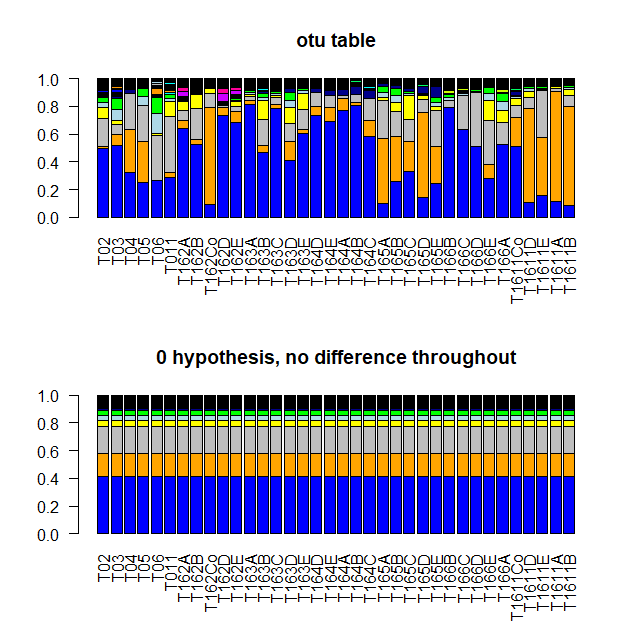

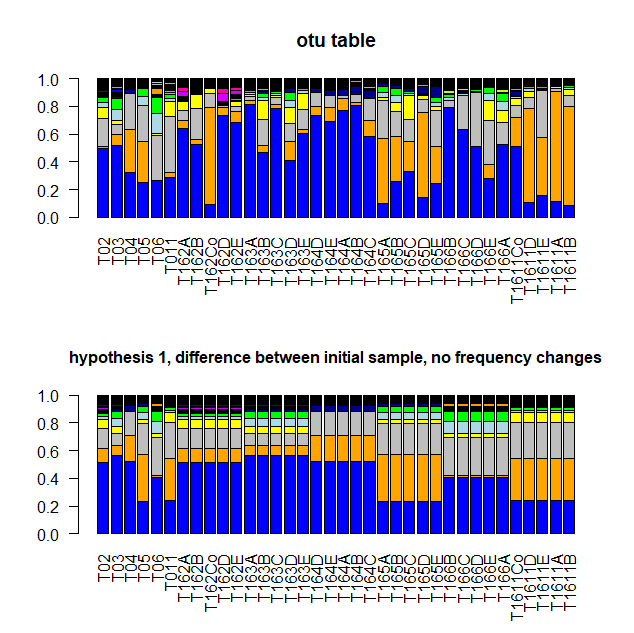

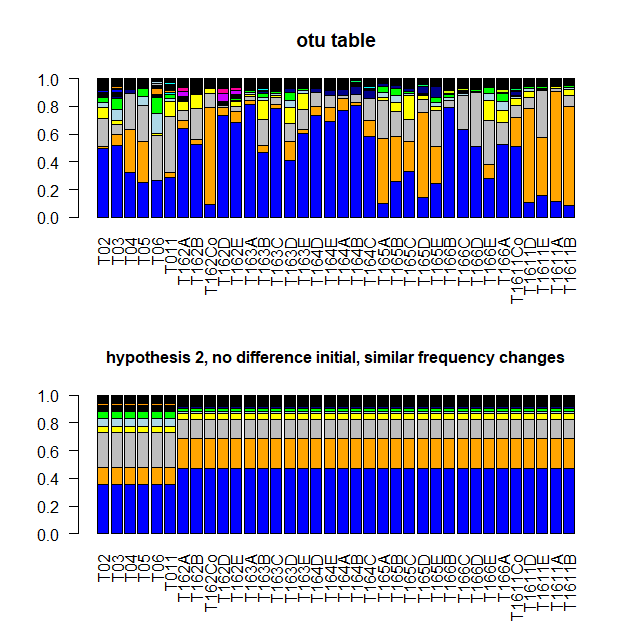


**T0**

**T16**

**S0**

**S1**

**S2**

**S3**

**S4**

**S5**

**Supplemental Figure S5.** Hypothetical community structures when allowing various factors in the construction of the communities from the total OTU pool. S0, all samples equal, no initial differences no frequency changes; S1, “Initial species diversity”, differences between sample, no changes in frequency; S2, “Time”, similar frequency changes, no differences initially; S3, “Initial species diversity + Time”, equal frequency changes and initial differences; S4, “Initial species diversity * Time”, different frequency changes and initial differences; S5, “Initial species diversity * Time + Stochasticity”, all samples at T16 a different distribution.
